# A Case of Pyorrhœa Alveolaris

**Published:** 1892-12

**Authors:** J. E. Cravens


AMERICAN JOURNAL

—OF—

DENTAL SCIENCE.

VOL. XXVI. BALTIMORE, DECEMBER, 1892. NO. 8.

ARTICLE I.

### A CASE OF PYORRHŒA ALVEOLARIS.

BY DR. J. E. CRAVENS.

Read at the American Dental Association, 1892.

The patient was a man of fifty years, robust and apparently in good health. The gums generally were red and angry-looking, bleeding easily and freely; expressed considerable pus from about the anterior eight teeth of each jaw, the festoons between which were swollen and of a violet color; "pockets" on the approximal faces of the pus-discharging cases. Treatment was begun on the 18th of July with the removal of the incrustations and trimming of the margins affected as far as possible, the "pockets" being washed out with hot water and carefully treated with dilute sulphuric acid (1-10). Pulverized sulphur, as a dentifrice, morning and night, was directed, and the use of soap and other alkalies in the mouth prohibited. After three days' daily treatment, but little improvement being manifest, the dilute sulphuric acid was substituted by a 10

per cent. solution of nitrate of silver, the washing with hot water being continued. No caustic action and no pain from the lunar caustic solution was observed. The next day no pus could be expressed, except from between the lower centrals and right inferior cuspid, and from between and around the right superior cuspid and lateral, which were the seats of the deeper pockets. Again washed out and treated with nitrate of silver. On the 25th congestion was gone, except slight violet tint over roots of inferior centrals, right inferior cuspid, and right superior lateral incisor, with only slight evidences of pus from these localities. Treated as before. On the 26th no pus except a minute quantity from one side of the right superior lateral incisor. Gum completely free from swelling. Again washed and treated. The next day no pus at any point; gums apparently healthy, and patient reported entire comfort. Again washed and treated as before. On the 29th no pus; "pockets" evidently filling up by adhesions. Again washed out with hot water, and treated with the nitrate of silver solution.

The writer noted that the nitrate of silver caused a slight brownish discoloration about the necks of the teeth treated, but it exhibited no caustic action whatever. He thought its astringent and antiseptic effects were emphasized by the thorough washing out of the "pockets" with hot water at each sitting, and he believed that the preliminary use of dilute sulphuric acid aided in removing small particles of calcareous matter from the "pockets" and stimulated granulation. The case appeared to be cured, and in an incredibly short time, though it would be under observation for some weeks.

Dr. W. C. Barrett, Buffalo, N. Y.: No disease within the province of dentistry calls for so great thought and reflection as pyorrhœa alveolaris. Its etiology has never been fully comprehended, or at least expressed. Remedies have been prescribed which are always successful in papers, and in practice almost always failures. At least that has been his experience, and also of those with whom he has talked. If pyorrhœa is a constitutional disorder, then mere local treatment is of no value. If it is merely a local disease, then local treatment should suffice for all ordinary cases. He has never found that local treatment would suffice in every case. In many cases topical remedies merely palliated the difficulty. What is wanted is something to eradicate the disease. By the exhibition of remedies he has been able to check the disease, but in six months or a year the patient will return with a new manifestation of it. That has been the almost invariable rule in the cases of that kind which he has attempted to treat. The remedy proposed in the paper is a cauterant. He knows of no therapeutic measure that seems to have value except to cauterize the tissues affected. Dr. Cravens' statement would indicate that the case described was a local manifestation and nothing else. It was only treated last month. Will it not return in three or six months? If it is simply a local manifestation of a constitutional disorder, there will be a return; but it would be interesting to know whether it has only been checked for the time being, and that is the speaker's opinion. He would like to hear from others, whether in such cases there is not a return of the disease with exaggerated force and virulency.

Dr. M. L. Rhein, New York, N. Y., would concede some of the points made by the last speaker, and others he would not agree to. From hearing the paper read he should have thought it was not a case of pure pyorrhœa, as distinguished from the false variety which results from bad hygienic management. One fault in these cases was often bad diagnosis. At the start it should be settled always whether the patient is in bad or good health, and this can only be done by thorough examination by a physician. Assuming that the patient was in health, he believes there was no pyorrhœa in this case. As to the etiology of pyorrhœa alveolaris, it is pretty fully established in his mind, and in the minds of many other practitioners, that the disease recognized as pyorrhœa alveolaris is either a sequence of some constitutional disorder, or an indication that constitutional disorder is present at the time; in other words, there is faulty nutrition. If the constitutional disease is curable, he can give a favorable prognosis of the pyorrhœa; if it is a sequence that is curable, he can give a more favorable prognosis. One special point should be to distinguish between the common form of what is sometimes called pyorrhœa, resulting from a badly kept mouth, and that which is a sequence of a constitutional disturbance. If we cannot determine this by ourselves, we can by consultation with a physician as to the condition of the patient. Many persons with this trouble have passed through his hands that never have been cured, and to whom he never promised a cure because he knew the case was hopeless, as where there was tuberculosis. Where he finds this in such grave form that the physician knows it to be incurable, it is useless to give a favorable prognosis.

Dr. W. H. Morgan, Nashville, Tenn.: As to the etiology of pyorrhœa alveolaris, observation more than any other theory had convinced him that the disease was constitutional, and in a large majority of cases inherited. Every case can be cured; just remove the teeth, and nature will do the rest. He did not know that it was ever cured except by the removal of the teeth.

Dr. A. W. Harlan, Chicago, Ill.: The subject under discussion is the treatment of a single case, and not the etiology of the disorder or a multitude of remedies for it. There is no objection to the use of dilute sulphuric acid as a preliminary step, because it contains 13 per cent. of the acid to 87 per cent. of water, and is then diluted ten times, so that when brought in contact with the soft tissues it does no damage. Nitrate of silver is an excellent remedy, and has been used for many years, but not in so concentrated a form as described by Dr. Cravens. It would be a powerful astringent, and the reason why it would check the flow of pus and close the "pockets" would be because of its astringent and stimulating effects. To say that the case was cured after eleven days' treatment is hardly credible, because it would be almost impossible, if one-half the depth of the roots were involved, for the tissue to be reproduced in that time. The use of hot water is an excellent idea; in a great many cases better than many other agents which are used. Dentists fail to utilize hot water as a therapeutic agent as much as they should, and Dr. Cravens has made a good point in calling attention to it for producing an effect on the gums. The speaker dilutes sulphuric acid with cinnamon water because he believes he gets a better effect than when he uses ordinary hydrant water.

Dr. Cravens: Dr. Barrett misunderstood the statement of the paper as to the effect of nitrate of silver solution. He had stated in the paper that he could observe no caustic effect. He could not agree with Dr. Rhein as to the constitutional origin of the trouble. Dr. Evans, of Paris, has seen so many cases of the disease associated with affections of the kidneys that he thought it was likely to be recognized as a manifestation of kidney trouble. It is also recognized on the other side of the ocean as a manifestation of gout. As to whether the case described in the paper was cured, he had simply expressed his opinion that it was cured.—*Cosmos.*

